# Supplementary material for: An open-source framework for large-scale, flexible evaluation of biomedical text mining systems
Source: J Biomed Discov Collab. 2008 Jan 29;3:1. doi: 10.1186/1747-5333-3-1 (PMC2276192; doi:10.1186/1747-5333-3-1)
Supplement: Additional file 1 — Evaluation platform source code. The additional file contains the software evaluation framework discussed in this paper. The most current release can be downloaded from SourceForge.net [1]. See the accompanying README file for instructions on installation and use. Also included in the distribution is a collection of UIMA wrappers for some commonly used BioNLP tools and annotated corpora. [file 1747-5333-3-1-S1.zip › bionlp_uima_repository-1.0.1/docs/api/edu/uchsc/ccp/util/nlp/mention/ComplexSlotMentionTypes.html]

ComplexSlotMentionTypes (Center for Computational Pharmacology Util/UIMA API)


|  |  |  |
| --- | --- | --- |
| **Overview** \| **Package** \| **Class** \| **Tree** \| **Deprecated** \| **Index** \| **Help** | |  |
| **PREV CLASS**   **NEXT CLASS** | **FRAMES**    **NO FRAMES**     **All Classes** |  |
| SUMMARY: NESTED \| FIELD \| CONSTR \| METHOD | DETAIL: FIELD \| CONSTR \| METHOD |  |


---


## edu.uchsc.ccp.util.nlp.mention Interface ComplexSlotMentionTypes

---

``` public interface ComplexSlotMentionTypes ```

---

| **Field Summary** | |
| --- | --- |
| `static String` | `BIOLOGICAL_SEQUENCE_ELEMENT_POSITION_IN_SEQUENCE` |
| `static String` | `BIOLOGICAL_SUBSEQUENCE_END_ELEMENT` |
| `static String` | `BIOLOGICAL_SUBSEQUENCE_START_ELEMENT` |
| `static String` | `COMPLEX_SLOT_MENTION_TYPE`             ComplexSlotMention Identifier - if this changes, Annotation DB must be adjusted accordingly |
| `static String` | `DELETION_DELETED_ELEMENT` |
| `static String` | `INSERTION_INSERTED_ELEMENT` |
| `static String` | `INSERTION_INSERTION_START` |
| `static String` | `SUBSTITUTION_MUTANT_ELEMENT` |
| `static String` | `SUBSTITUTION_WILDTYPE_ELEMENT` |

**Field Detail**

### COMPLEX\_SLOT\_MENTION\_TYPE

```
static final String COMPLEX_SLOT_MENTION_TYPE
```

:   ComplexSlotMention Identifier - if this changes, Annotation DB must be adjusted accordingly

    **See Also:**: Constant Field Values

---


### SUBSTITUTION\_MUTANT\_ELEMENT

```
static final String SUBSTITUTION_MUTANT_ELEMENT
```

**See Also:**: Constant Field Values

---


### SUBSTITUTION\_WILDTYPE\_ELEMENT

```
static final String SUBSTITUTION_WILDTYPE_ELEMENT
```

**See Also:**: Constant Field Values

---


### DELETION\_DELETED\_ELEMENT

```
static final String DELETION_DELETED_ELEMENT
```

**See Also:**: Constant Field Values

---


### INSERTION\_INSERTED\_ELEMENT

```
static final String INSERTION_INSERTED_ELEMENT
```

**See Also:**: Constant Field Values

---


### INSERTION\_INSERTION\_START

```
static final String INSERTION_INSERTION_START
```

**See Also:**: Constant Field Values

---


### BIOLOGICAL\_SEQUENCE\_ELEMENT\_POSITION\_IN\_SEQUENCE

```
static final String BIOLOGICAL_SEQUENCE_ELEMENT_POSITION_IN_SEQUENCE
```

**See Also:**: Constant Field Values

---


### BIOLOGICAL\_SUBSEQUENCE\_START\_ELEMENT

```
static final String BIOLOGICAL_SUBSEQUENCE_START_ELEMENT
```

**See Also:**: Constant Field Values

---


### BIOLOGICAL\_SUBSEQUENCE\_END\_ELEMENT

```
static final String BIOLOGICAL_SUBSEQUENCE_END_ELEMENT
```

**See Also:**: Constant Field Values


---


|  |  |  |
| --- | --- | --- |
| **Overview** \| **Package** \| **Class** \| **Tree** \| **Deprecated** \| **Index** \| **Help** | |  |
| **PREV CLASS**   **NEXT CLASS** | **FRAMES**    **NO FRAMES**     **All Classes** |  |
| SUMMARY: NESTED \| FIELD \| CONSTR \| METHOD | DETAIL: FIELD \| CONSTR \| METHOD |  |


---
